# Proximal Femoral Fracture in Hip Arthrodesis Treated with Double Reconstruction Plates

**DOI:** 10.1155/2017/5246080

**Published:** 2017-06-11

**Authors:** Shunsuke Asakawa, Takeo Mammoto, Atsushi Hirano

**Affiliations:** Department of Orthopaedic Surgery and Sports Medicine, Tsukuba University Hospital Mito Clinical Education and Training Center, Mito Kyodo General Hospital, University of Tsukuba, 3-2-7 Miya-Machi, Mito, Ibaraki 310-0015, Japan

## Abstract

We present a rare clinical case of a 90-year-old female who sustained a proximal femoral neck fracture following long-standing hip arthrodesis. Since the fracture occurred relatively proximally and involved the pelvis, double-plate fixation was chosen to achieve rigid fixation. The reconstruction plate was placed at the posterior and anterior columns individually through single vertical incision. She was treated successfully, and she attained preinjury activity level. Proximal femoral fractures in arthrodesed hips need to be recognized as a fracture between the pelvis and femur. Rotational stress from the trunk and lower extremity requires rigid fixation to minimize the increase of displacement and the risk for nonunion.

## 1. Introduction

The number of patients who suffer from fractures around the hip has been increasing in aging society. A proximal femoral fracture is one of the most common fractures. There are a lot of surgical options that have been developed in recent years to treat these and other similar fractures; however, a proximal femoral fracture occurring in an arthrodesed hip is uncommon. In such a case, there are no definitive treatment strategies.

Here, we report a case of a proximal femoral neck fracture following long-standing hip arthrodesis, where good clinical results were achieved when treated with double reconstruction plates.

## 2. Case Report and Surgical Technique

A 90-year-old female was admitted to our hospital complaining of pain around the left hip after a fall. She had a history of undergoing hip arthrodesis surgery following onset of tuberculosis in her 40s. Although her left hip was immovable with the affected leg length appearing shortened, she had still been able to walk long distances using a cane prior to the injury.

**Figure 1:**
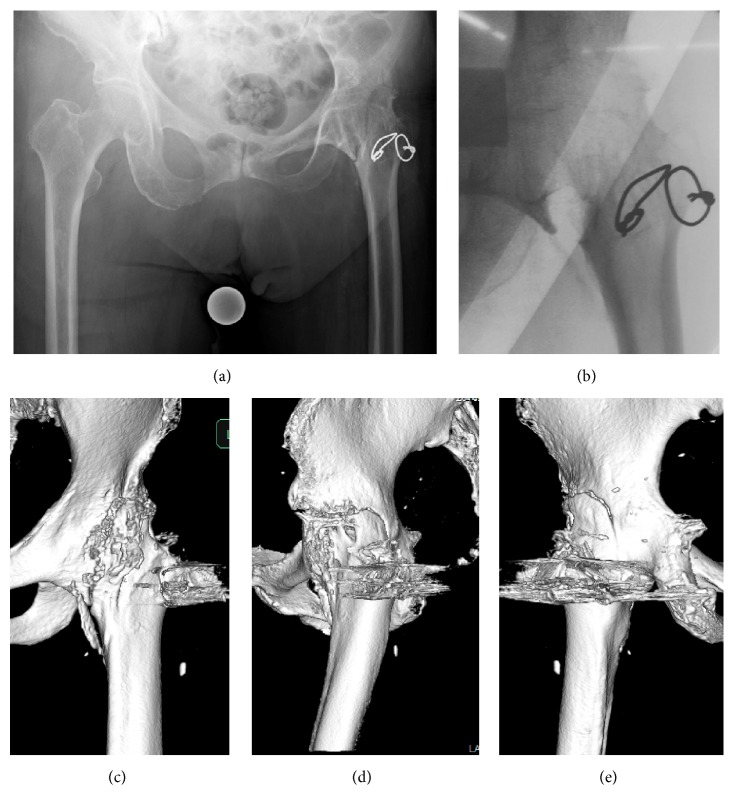
Preoperative images of the hip. (a) Radiograph of the anteroposterior (AP) view. (b–d) 3D-CT: (b) AP, (c) lateral, and (d) posterior-anterior (PA) views of the hip. Proximal femoral fracture in arthrodesed hip. Fracture line extends to pelvis. (e) Intraoperative image by intensifier. Instability of fracture site was confirmed and the displacement was increased with hip adduction.

Radiographs showed both proximal femoral and pelvic fractures. The fracture line started from the ilium, involving the original femoral head, and ended at the basicervical part of the femoral neck (Figures 1(a)–1(e)). The fracture type appeared to be a vertical fracture. A computed tomography (CT) scan of the pelvis revealed ankylosis between the acetabulum and proximal femoral head. The structural border could not be identified between the pelvic bone and femur head. Surrounding tissues including subcutaneous tissue and the muscles were observed to be severely atrophied in comparison with those of the opposite side. Although the amount of displacement was approximately 2 mm, the vertical fracture was believed to be unstable and had a risk of displacement worsening during any increase in weight bearing. At this point, we determined the appropriate surgical intervention.

Although several surgical options have been reported previously in the literature, the proper techniques for certain distinctive scenarios have not yet been well described. In this case, double-plate fixation was thought to be the right surgical option to achieve rigid fixation with pelvis.

Surgery was performed under general anesthesia. Displacement was increased with passive hip adduction, and obvious instability was confirmed (Figure 1(b)).

**Figure 2:**
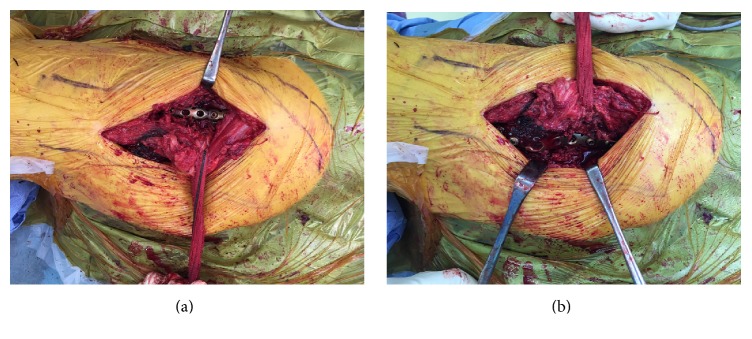
Single incision approach was useful for reduction and fixation. (a) Anteromedial plate was positioned, retracting gluteus medius posteriorly. (b) Posterior plate was positioned, retracting gluteus medius anteriorly.

The patient was placed in the right lateral decubitus position with the injured side abducted. The lateral approach with a single vertical incision was chosen. After splitting the atrophied gluteus maximus, the fascia of the gluteus medius was exposed. The anterior border of the gluteus medius was identified, and the tensor fasciae latae was retracted anteriorly. The gluteus medius was elevated to access the anteromedial part of fracture. The posterior part of fracture line was then similarly exposed by retracting the gluteus medius anteriorly (Figures 2(a) and 2(b)).

**Figure 3:**
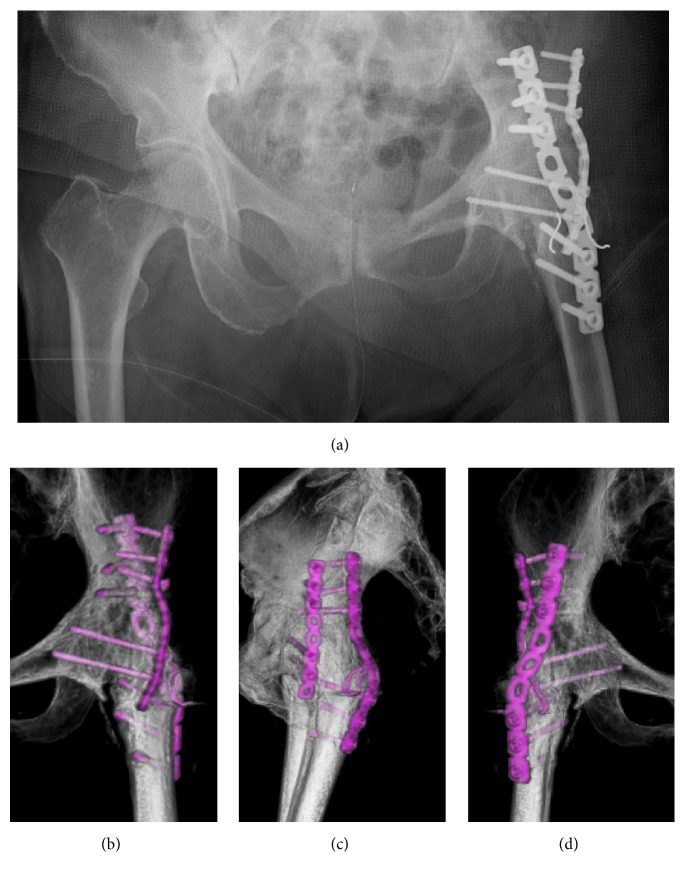
Postoperative images of the hip radiograph and 3D-CT of hip. (a) Radiograph of AP view. (b–d) 3D-CT: (b) AP, (c) lateral, and (d) PA views of the hip. Double-plate fixation positioned at the anteromedial and posterior aspects of the fracture site.

A reconstruction plate was placed at the posterior and anterior columns with anatomical bending. Screws were inserted in a bicortical manner. Distal screws were inserted to the original femoral head through the fracture line (Figure 3). Appropriate reduction and rigid fixation were confirmed under the image intensifier (Figures 3(a)–3(d)).

**Figure 4:**
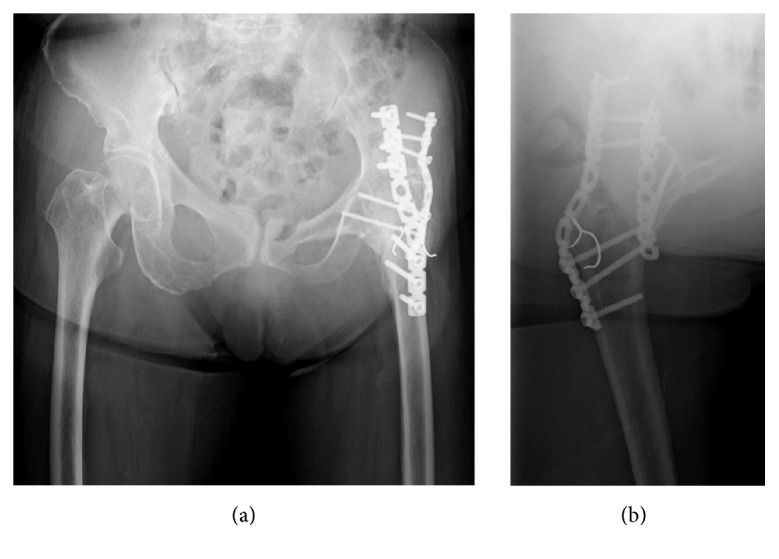
Follow-up radiographs at 10 months after surgery. (a) AP view and (b) lateral view. Bone union of fracture site was revealed with no displacement and no implant complication.

Postoperatively, the patient was immobilized with a hip spica cast for 4 weeks. Then, partial weight bearing was initiated, and full weight bearing was allowed beginning at 8 weeks after surgery. The fracture appeared united on the radiographs examined 3 months after surgery. In the follow-up 10 months after surgery, radiographs and CT revealed bone union without fracture site displacement and no implant complication (Figures 4(a) and 4(b)). She returned her ADL activities as preinjury levels.

## 3. Discussion

The proximal femur is the anatomic region frequently involved in fragile fractures, while fractures occurring in an arthrodesed hip are relatively rare. Sponseller et al. assessed long-term follow-up in hip arthrodesis patients and revealed that only 2 of 53 patients sustained a femoral fracture [1]. Femoral neck fracture in cases of osteoarthritic hip is uncommon because of the proliferation of the trabecular bone and the alternation of loading stress distribution [2]. Due to the rare occurrence of proximal femoral fracture in an arthrodesed hip, appropriate management methods are not currently established for this type of fracture.

Nonsurgical treatments might provide excellent clinical results for those with nondisplaced fractures. However, previous studies recommend surgical intervention for the following reasons: first, similar to cases of common proximal femoral fractures, it is important to shorten the periods of bed rest to avoid functional weakness. In addition, rotational stress from the trunk and long lever arm of the lower extremity make it difficult to maintain stability enough to achieve bone union [3]. Additionally, vertical fracture lines generate shear stress between the pelvic and femoral bones. For these reasons, we thought that surgical treatment would lead to better results than conservative treatment options.

Surgical options are mainly divided into two techniques: total hip replacement (THR) and open reduction and internal fixation (ORIF). THR for hip arthrodesis is expected to bring some benefits, including improvement in range of motion and leg length discrepancy [4]. However, inserting an acetabular cup into the appropriate position is difficult because of pelvic deformity and secondary changes of the lumbar spine [5]. Malposition of an acetabular cup also increases the risk of postoperative dislocation. Furthermore, the atrophy of gluteus muscles and surrounding tissues could increase the risk of postoperative dislocation [4, 6]. THR for hip arthrodesis is also characterized by a significantly higher occurrence rate of postoperative infection and nerve injury, as compared to THR for hip osteoarthritis [4]. Moreover, reactivation in hip arthrodesis after tubercular arthritis has been reported, even after a quiescent period of more than 30 years [7].

ORIF does not provide either improvement of range of motion or leg length discrepancy. However, this surgical technique is relatively easier to perform and is less invasive and less fraught with complications, as compared with THR. Therefore, ORIF is indicated for those who are less likely to tolerate invasive surgery comorbidities or elderly people. Our patient was elderly, and her activity level before injury was not high, so we decided to perform ORIF to restore her preinjury activity levels with the less invasive treatment. Various other surgical options have been previously reported including interlocking nail, dynamic hip screw (DHS) and plates, or cannulated cancellous screw (CCS) [3, 4, 8–10].

In the case of arthrodesed patients, intertrochanteric fracture is the most frequent, while those involving the femoral neck or those that are more proximal are uncommon. When the proximal fragment is large enough to place long screws, rigid fixation might be achieved via interlocking nail, DHS, or multiple screws [3, 4, 8, 9]. However, in our case, the fracture occurred relatively proximally, involving the pelvis. When the proximal fragment is smaller, use of interlocking nail, DHS, or CCS might be inadequate to maintain proper positioning against rotational or vertical stress. Therefore, double-plate fixation was chosen to get rigid fixation in this case.

To our knowledge, three other studies report treatment with this method. All the cases reported in these studies suffered intertrochanteric lesion and plate fixation was chosen, which achieved a satisfactory outcome [6, 8, 9]. Manzotti et al. positioned the double plates to the anterior and lateral aspects of the acetabulum and femur. There were no detailed reports regarding the surgical approach or process of the exposure to the fracture site [8]. Darwish and Haddad used a single plate in combination with cannulated screws, placed through the lateral approach. These screws were inserted perpendicularly to the fracture plane, and the locking plate was adopted additionally as a neutralization plate [6]. Okamoto et al. placed a single plate laterally through the lateral approach [9].

In our case, double plates bridging from the femur to pelvis were positioned at the anteromedial and posterior aspects through a single lateral incision. With this incision, it is easy to expose the fracture site from the anterior and posterior borders of the gluteus medius individually. This surgical approach is useful for proximal femoral fractures in cases of long-standing hip arthrodesis because it minimizes the damage to the gluteus muscles and enables double-plate fixation with no additional incision.

## 4. Conclusion

Proximal femoral fractures in arthrodesed hip need to be recognized as fractures between the pelvis and femur. Rotational stress from the trunk and lower extremity requires rigid fixation to minimize the increase of displacement and the risk for nonunion.
